# Early Kinetics of Intestinal Infection and Immune Responses to Two *Toxoplasma gondii* Strains in Pigs

**DOI:** 10.3389/fcimb.2020.00161

**Published:** 2020-04-16

**Authors:** Mizanur Rahman, Bert Devriendt, Malgorzata Jennes, Ignacio Gisbert Algaba, Pierre Dorny, Katelijne Dierick, Stéphane De Craeye, Eric Cox

**Affiliations:** ^1^Laboratory of Immunology, Department of Virology, Parasitology and Immunology, Faculty of Veterinary Medicine, Ghent University, Merelbeke, Belgium; ^2^Sciensano, National Reference Center for Toxoplasmosis, Infectious Diseases in Humans, Brussels, Belgium; ^3^Department of Biomedical Sciences, Institute for Tropical Medicine, Antwerp, Belgium; ^4^Laboratory of Parasitology, Department of Virology, Parasitology and Immunology, Faculty of Veterinary Medicine, Ghent University, Merelbeke, Belgium

**Keywords:** *Toxoplasma gondii*, pigs, magnetic capture-qPCR, intestine, IFNγ

## Abstract

*Toxoplasma gondii* is an obligate intracellular parasite, able to infect all homeothermic animals mostly through ingestion of (oo)cysts contaminated food or water. Recently, we observed a *T. gondii* strain-specific clearance from tissues upon infection in pigs: while the swine-adapted LR strain persisted in porcine tissues, a subsequent infection with the human-isolated Gangji strain cleared parasites from several tissues. We hypothesized that intestinal immune responses shortly after infection might play a role in this strain-specific clearance. To assess this possibility, the parasite load in small intestinal lymph node cells and blood immune cells as well as the IFNγ secretion by these cells were evaluated at 2, 4, 8, 14, and 28 days post oral inoculation of pigs with tissue cysts of both strains. Interestingly, at day 4 post inoculation with the LR strain the parasite was detected by qPCR only in the duodenal lymph node cells, while in the jejunal and ileal lymph node cells and PBMCs the parasite was detected from day 8 post inoculation onwards. Although we observed a similar profile upon inoculation with the Gangji strain, the parasite load in the examined cells was much lower. This was reflected in a significantly higher *T. gondii*-specific serum IgG response in LR compared to Gangji infected pigs at day 28 post inoculation. Unexpectedly, this was not reflected in the IFNγ secretion upon re-stimulation of the cells where almost equal IFNγ secretion was observed in both groups. In conclusion, our results show that *T. gondii* first enters the host at the duodenum and then probably disseminates from this site to the other tissues. How the early immune response influences the clearance of parasite from tissues needs further study.

## Introduction

The obligate intracellular protozoan parasite *Toxoplasma gondii* causes toxoplasmosis in all homeothermic animals which is life-long and often asymptomatic. In immunocompromised patients, toxoplasmosis can be fatal, while in pregnant women congenital toxoplasmosis might result in fetal and neonatal mortality, neurologic abnormalities, chorioretinitis, and other symptoms reviewed by Torgerson and Mastroiacovo (2013). In 2013, the global annual incidence of congenital toxoplasmosis was calculated to be equivalent to a disease burden of 1.20 million disability-adjusted life years (Torgerson and Mastroiacovo, 2013).

*T. gondii* is an important foodborne pathogen and natural human infection commonly results from the ingestion of raw or undercooked meat containing tissue cysts or food/drinks contaminated with sporulated oocysts. After ingestion, the cyst wall protects the parasites from the gastric pH and ensures passage to the small intestine where they excyst upon contact with bile salts and trypsin (Dubey, 1997; Dubey et al., 1998). Upon excystation, initial bradyzoite invasion and replication takes place at the tips of the villi and subsequently results in the release of tachyzoites into the lumen to colonize neighboring villi and in the lamina propria to disseminate in the host (Dubey, 1997; Dubey et al., 1998; Coombes et al., 2013; Verhelst et al., 2014). In mice, the proximal jejunum seems to be the preferred replication site (Gregg et al., 2013). Transmigration through the intestinal epithelial cells possibly involves an unusual form of gliding motility, paracellular transmigration, penetration of the apical cell membrane, and use of immune cells in a Trojan horse-like mechanism (Barragan et al., 2005; Courret et al., 2006; Gregg et al., 2013; Jones et al., 2017). In response to the infection, neutrophils are rapidly recruited to the infected sites and are subsequently invaded by progeny *T. gondii* egressing from the infected epithelial cells (Coombes et al., 2013). Part of the parasite invaded neutrophils migrate to the lumen and facilitate re-infection of neighboring villi, while the rest spreads to the draining lymph nodes and enters the blood circulation to disseminate to other organs. In addition, many other cell types can be invaded by *T gondii* including dendritic cells (DCs), mononuclear phagocytes, NK cells, and lymphocytes (Courret et al., 2006; Lambert et al., 2006; Coombes et al., 2013). Interestingly, *T. gondii* within immune cells can sense the arrival of these cells at the target organs via adhesion to certain surface molecules on capillary endothelial cells, e.g., CD162 on lung endothelial cells, and immediately egress the immune cells to infect target organs (Baba et al., 2017).

However, most of these mouse data are inapposite for humans and warrant further investigation in relevant large animal models. For example, upon *T. gondii* infection, murine DCs undergo maturation in response to the parasite antigen profilin via TLR11 and TLR12. However, in humans and pigs TLR11 and TLR12 are non-functional pseudogenes (Tosh et al., 2016). Therefore, to elicit an immune response they rely on phagocytosis of tachyzoites and subsequent recognition of *T. gondii* RNA and DNA via TLR7,−8, and−9 (Forsbach et al., 2008; Ishii et al., 2008; Andrade et al., 2013; Weidner et al., 2013; Betancourt et al., 2019).

In humans there is still a major knowledge gap between the early infection of the small intestinal epithelium and the dissemination of the parasite to other organs to establish tissue cysts as well as the associated immune responses. To address this gap pigs may be a particularly interesting model. Indeed, the immune system as well as the gut physiology of pigs closely resembles that of humans (Meurens et al., 2012). In addition, pigs are susceptible to *T. gondii* infection. However, also in pigs thorough knowledge is lacking on the initial events during infection of the small intestine, the subsequent dissemination and the associated small intestinal immune responses.

*T. gondii* virulence not only differs between animals, but also among *T. gondii* strains, which in Europe belong to three major genotypes (e.g., type I, II, and III) based on the DNA sequence of multilocus analysis. Genotype II is the most prevalent genotype in livestock species and humans (Howe and Sibley, 1995; Dubey, 2010). Our previous results showed that different genotypes trigger variable immune responses in pigs (Jennes et al., 2017). A genotype II strain (IBP LR) elicited stronger IFNγ^+^ T cell responses as compared to a hybrid genotype I/II strain (IBP Gangji). These LR strain-specific immune responses seemed to play a role in the clearance of tissue cysts upon infection of pigs with the Gangji strain (Jennes et al., 2017). However, our understanding on how different genotypes impact the early infection stages of *T. gondii* is incomplete. In this study, we aimed to investigate the early infection kinetics, antibody and IFNγ response for both strains. The latter since IFNγ seems to play a crucial role in clearance of the parasite from infected hosts (Jennes et al., 2017).

## Materials and Methods

### Animals and Ethics Statement

Thirty-six four-week-old piglets (Belgian Landrace x large white) were obtained from a high health-status farm in Belgium and transported to the Faculty of Veterinary Medicine, Ghent University, where the piglets were housed in isolation units and were given *ad libitum* access to feed and water. The animal procedures were approved by the Ethical Committee (EC) of the Faculty of Veterinary Medicine and the Faculty of Bioscience Engineering, Ghent University (EC 2009/149) and by the EC of Sciensano, Belgium (176 20140704-01).

### *T. gondii* Strains

The *T. gondii* IPB LR and Gangji strains were used to inoculate pigs. The LR strain was originally isolated from pigs and belongs to genotype II, which is commonly present in the European pig population. It is less virulent in mice than the hybrid genotype I/II Gangji strain, which is highly virulent in mice (Dubey et al., 2012; Jennes et al., 2017). The latter strain was isolated from the placenta of a pregnant woman having a congenitally infected baby (Ajzenberg et al., 2010). Both strains were isolated and maintained at the National Reference Laboratory for Toxoplasmosis, Sciensano, Brussels, Belgium by passage in Swiss Webster female mice (EC: 176 20140704-01). For oral inoculation, tissue cysts of both strains were harvested from infected mouse brain tissue, counted by phase-contrast microscopy and suspended in sterile phosphate buffered saline (PBS) at a concentration of 1,000 tissue cysts/ml (Jennes et al., 2017).

### Antigen Preparation

*T. gondii* lysate antigen (TLA) was prepared from tachyzoites of the RH-strain as previously described (Jongert et al., 2007). The TLA antigens were concentrated and dialyzed with Amicon® filter units (cut-off = 10 kDa) and finally diluted in PBS. The bicinchoninic acid (BCA) reaction (Thermo Scientific Pierce BCA protein Assay Kit, Erembodegem, Belgium) was used to determine the protein concentration and upon filter sterilization with low protein binding filters (0.22 μm, Millex-GV) TLA was stored at −20°C until further use.

### Experimental Set-Up

Upon arrival, 36 four-week-old piglets (Belgian Landrace x large white) were confirmed to be *T. gondii* seronegative by the modified agglutination test (ToxoScreen DA, Biomérieux, Capronne, France) and an immunofluorescence test (Toxo-Spot IF, Biomérieux) as described previously (Verhelst et al., 2015). The piglets were randomly selected with taking into account their gender and divided into three groups: control (*n* = 6), LR group (*n* = 15), and Gangji group (*n* = 15).

**Figure 1:**
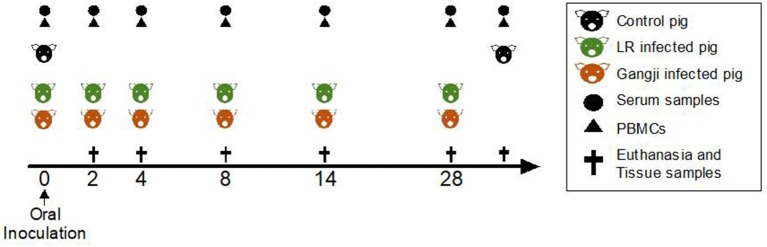
Scheme of the study. Experiment day is indicated as numbers (0–35). TLA-ELISA: *T. gondii* lysate antigen-specific enzyme-linked immunosorbent assay. PBMCs, peripheral blood mononuclear cells. MCqPCR, magnetic capture real time quantitative polymerase chain reaction.

At day zero (D0) the piglets were inoculated with 6,000 tissue cysts of the *T. gondii* IPB LR strain or Gangji strain, respectively (Figure 1). Blood was sampled at the indicated time points to evaluate serum antibody responses by ELISA and the presence of antigen-specific immune cells in an *in vitro* recall assay. At 2, 4, 8, 14, and 28 days post infection, 3 LR strain infected and 3 Gangji strain infected piglets, and at day 0 and day 35, 3 control animals, were euthanized by injecting sodium pentobarbital (20%, 0.125 ml/kg bodyweight; Nembutal; Sanofi) and following exsanguination lymph nodes and tissue samples were collected to assess the parasite load.

### *In vitro* Antigen Recall Assay

Peripheral blood MCs (PBMCs) were isolated from heparinized blood samples with density gradient centrifugation as described (Verhelst et al., 2014). Mononuclear cells (MCs) from the duodenum, jejunum, ileum and mediastinal lymph nodes were harvested in RPMI (1640; Gibco, Merelbeke, Belgium) supplemented with 100 U/ml penicillin and 100 μg/ml streptomycin (P/S; Gibco) as described (Verhelst et al., 2011). The cell suspension was cleared with a 70 μm cell strainer (Corning, USA) and erythrocytes were lysed in lysis buffer (9:1 of 0.83% w/v NH_4_Cl and 2.06% w/v Tris (C_4_H_11_NO_3_), pH 7.2). After washing in PBS + 1 mM EDTA and centrifugation at 380 g for 10 min at 18°C, the pelleted cells were resuspended in complete leukocyte medium [RPMI 1640 supplemented with 10% fetal calf serum (FCS, Greiner Bio-One, Belgium), 292 μg/ml l-glutamin (Gibco), 100 IU/ml penicillin and 100 μg/ml streptomycin (P/S; Gibco), 100 mM non-essential amino acids (Gibco) and 100 μg/ml kanamycin (Gibco)] at a final concentration of 1 × 10^7^ cells/ml.

The MCs were seeded in sterile 96-well flat bottom cell culture plates (Greiner Bio-One) at 1 × 10^6^ cells/well in complete leukocyte medium. After an initial incubation of 1 h, the plates were incubated with 20 μg/ml TLA or medium for 72 h at 37°C in a humidified atmosphere with 5% CO_2_. After incubation, the cell-free supernatant was collected and stored at −20°C until analysis of the IFNγ concentration by ELISA.

### DNA Extraction and qPCR

DNA was extracted from the isolated mononuclear cells (1.1 × 10^7^ cells) using the QIAamp® DNA Mini kit (Qiagen GmbH, Hilden, Germany) according to the manufacturer's instructions.

The *T. gondii* DNA was quantified by a duplex real-time TaqMan quantitative PCR analysis as described (Gisbert Algaba et al., 2017). Briefly, 10 μl of the DNA extract was tested in a final reaction volume of 25 μl containing 12.5 μl of ExTaq 2 × probe mix (Takara, Saint-Germain-en-Laye, France), 400 nM of primers T2 and T3, 200 nM of primers VF1 and VR1, 66 nM of *Toxoplasma* probe and 40 nM of r18S probe. Each DNA sample was tested twice in each PCR run: one to check the presence of *T. gondii* DNA and cellular r18S and the other one with only the primers and probes to amplify the *T. gondii* target. The real-time PCR was performed with the following cycling program: 3 min at 95°C, followed by 41 cycles of 15 s at 95°C and 20 s at 60°C on a BioRad CFX 96 thermocycler (Hercules, California, USA). In each run, non-template controls were included. The qPCR results were analyzed to obtain the quantification cycle (Cq) values using the BioRad CFX manager software. For the quantification of *T. gondii* parasite DNA, a standard curve was generated from the Cq values of a 10 fold diluted known number of tachyzoites, i.e., *T. gondii* RH strain (Gisbert Algaba et al., 2017).

### TLA ELISA

Blood samples were collected in vacuum tubes (Vacutest KIMA, Italy) from the vena jugularis and were allowed to clot at room temperature for 30 min. Subsequently, serum samples were collected upon centrifugation at 15,000 g for 10 min, aliquoted and stored at −20°C until further use. TLA-specific serum IgG responses were evaluated by ELISA as described (Verhelst et al., 2015). Briefly, 96-well microliter plates were coated with TLA (5 μg/ml, Microbix Biosystems, Canada), serial diluted serum samples were added and detected with HRP-conjugated anti-porcine IgG (Bethyl Laboratories Inc., Montgomery, Texas, USA) and ABTS, i.e., 2,2′-azino-bis (3-ethylbenzothiazoline-6-sulphonic acid) as a substrate. On each plate previously collected sera from positive and negative control animals, as established by IgM and IgG immunofluorescence assay (IFA), were included. After 45 min incubation with substrate at 37°C, the absorbance at 405 nm was measured with a microplate reader (TECAN Spectra Fluor, Tecan Group Ltd., Männedorf, Switzerland) and the obtained data were analyzed in GraphPad Prism 6 software. Serum samples from infected animals were considered positive when exceeding the cut-off value (= mean OD_405_ negative controls + 3× the standard deviation). Antibody titers were calculated as the inverse of that dilution with a signal above the cut-off value.

### IFNγ ELISA

IFNγ secretion was determined in the supernatant (1/10, 1/20, and/or 1/50 dilution) of MCs cultured in medium or stimulated with antigens as described above with a sandwich ELISA using the swine-specific IFNγ antibody pair kit (ThermoFisher). The IFNγ concentration in the supernatants was calculated from a regression line (4-parameter curve fit) of serially diluted standard using the DeltaSoft JV 2.1.2 software. The limit of detection of this ELISA was 12.3 pg/ml.

### Magnetic Capture qPCR

To assess the *T. gondii* load in lungs and heart, these tissues were collected upon euthanasia and the parasite load was determined via an ISO 17025 validated magnetic capture qPCR (MCqPCR) as described (Gisbert Algaba et al., 2017). This technique combines the magnetic isolation of *T. gondii*-specific DNA from large tissue samples (>100 g) with the sensitivity of qPCR. It has an improved sensitivity of 94.12% as compared to another MCqPCR method (Opsteegh et al., 2010).

The samples with a quantification cycle (Cq) crossing the threshold were considered positive for *T. gondii*, while samples with no Cq for the *T. gondii* target, but Cq of the not competitive internal amplification control were considered negative. The detection limit of this method is 65.4 parasites per 100 g of tissue sample. For each round of samples, a positive control with a known number of parasites was included to correct for possible deviations due to manipulation errors. The number of parasites (n° p) was calculated according to the following formula:

$$\begin{array}{l} \log_{10}\left(n^{{}^{\circ}}p\right)=\;\frac{\text{C}{\text{q}}_{\text{value}}-44.75}{-3.0788}\; \end{array}$$

The formula resulted from a standard curve established with known concentrations of parasites ranging from 100 to 10^5^ spiked in 100 g of muscle tissue samples or in 50 g of brain tissue (Gisbert Algaba et al., 2017). Log_10_(n° p) represents the log10-transformed parasitic load, while the Cq_value_ represents the point on the exponential amplification curve crossing the threshold.

### Data Analysis

The antibody responses, IFNγ response and *T. gondii* parasite load in tissue samples of the different groups are presented as mean ± SD. Data were analyzed in GraphPad Prism 6 software with the Friedman test and a *post hoc* analysis via Dunn's test. In all analyses p<0.05 was considered statistically significant.

## Results

### The Parasite Load in Gut Lymph Node Cells Indicates That *T. gondii* Initially Enters the Host via the Duodenum

**Figure 2:**
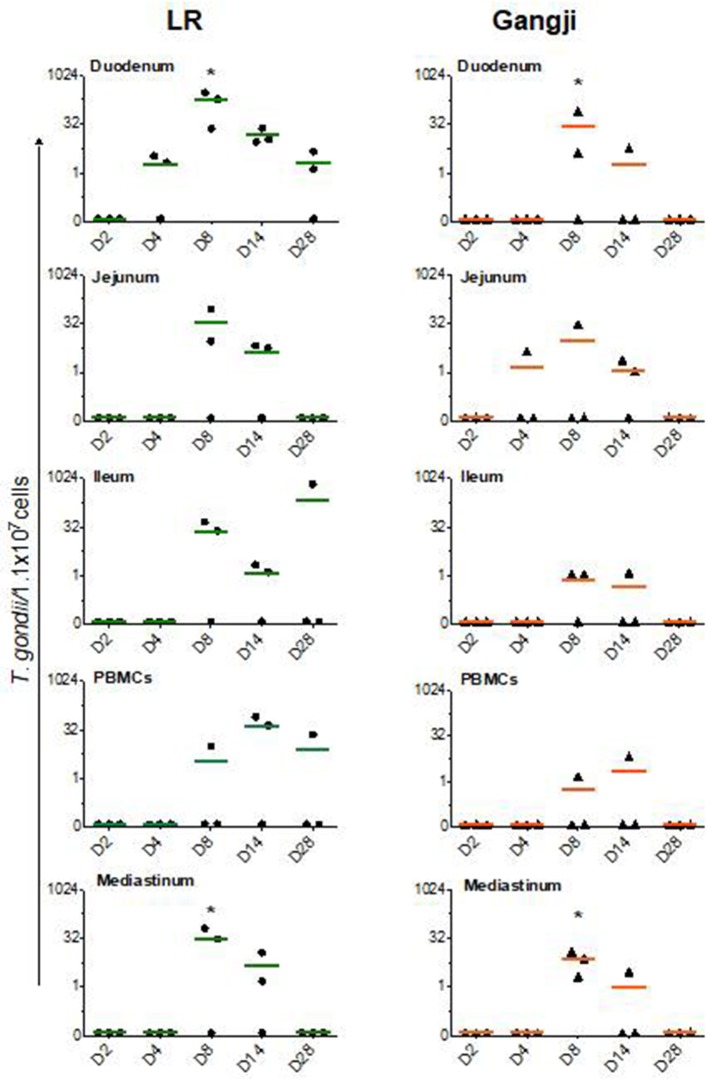
*T. gondii* DNA load in immune cells isolated from lymph nodes and blood. The data are presented as the number of *T. gondii* parasites/1.1 × 10^7^ cells. LN, Lymph node. The horizontal line represents the mean, *significantly different than D2, *P < 0.05.

Since *T. gondii* migrate to the mesenteric lymph nodes for rapid dissemination to the target organs via the blood circulation, we assessed the parasite load in immune cells isolated from small intestinal mesenteric lymph nodes (LNs) to trace the initial entry site of the parasite. In the LR group, the parasite load reached its maximum at D8 in all mesenteric lymph nodes as compared to PBMCs in which the parasite load peaked at D14. However, the parasite load in duodenal, jejunal and ileal LNs differed considerably (Figure 2) (Table 1). *T. gondii* DNA in mononucleated cells (MCs) of duodenal LNs was first detected at day 4 post inoculation, which further increased to reach its maximum at D8 (*P* = *0.0196*) and then steadily dropped. In contrast, in jejunal and ileal LNs *T. gondii* DNA was only first detected at day 8 post inoculation, and subsequently decreased following a similar pattern as the duodenal LNs. On the other hand, in the Gangji group, the *T. gondii* DNA load in the MCs of duodenal, jejunal and ileal LNs reached its peak at D8 (*P* = *0.045*) and dropped below the detection limit at D28 (Figure 2). Interestingly, in duodenal mesenteric LN of this group, the parasite load was below the detection limit at D4.

**Table 1:** Parasite load in mononuclear immune cells isolated from lymph nodes and blood as determined by qPCR.

**Group**	**Experiment Day**	**Animals**	***T. gondii*** **load**				
			**LN duodenum**	**LN jejunum**	**LN ileum**	**PBMCs**	**LN mediastinum**
LR		1	0	0	0	0	0
	D2	2	0	0	0	0	0
		3	0	0	0	0	0
		Average	**0**	**0**	**0**	**0**	**0**
		4	0	0	0	0	0
	D4	5	2.09	0	0	0	0
		6	3.35	0	0	0	0
		Average	**1.81**	**0**	**0**	**0**	**0**
		7	299.91	88.66	24.59	0	64.50
	D8	8	187.86	0	46.12	0	0
		9	23.17	9.10	1	10.16	30.26
		Average	**170.31**	**32.59**	**23.90**	**3.39**	**31.58**
		10	23.81	5.70	2.21	79.90	11.43
	D14	11	10.75	0	0	43.69	0
		12	8.98	6.61	1.33	0	1.51
		Average	**14.51**	**4.10**	**1.18**	**41.20**	**4.31**
		13	0	0	0	0	0
	D28	14	1.33	0	496.5	0	0
		15	4.54	0	0	22.41	0
		Average	**1.95**	**0**	**165.50**	**7.47**	**0**
Gangji		16	0	0	0	0	0
	D2	17	0	0	0	0	0
		18	0	0	0	0	0
		Average	**0**	**0**	**0**	**0**	**0**
		19	0	0	0	0	0
	D4	20	0	4.44	0	0	0
		21	0	0	0	0	0
		Average	**0**	**1.48**	**0**	**0**	**0**
		22	4.33	0	1.10	1	7.67
	D8	23	81.10	29.70	1.14	1.51	12.05
		24	0	0	0	0	2.13
		Average	**28.48**	**9.90**	**0.75**	**0.83**	**7.28**
		25	5.72	2.31	0	1	2.96
	D14	26	0	1.09	1.27	0	0
		27	0	0	0	6.88	0
		Average	**1.91**	**1.13**	**0.42**	**2.29**	**0.99**
		28	0	0	0	0	0
	D28	29	1	0	0	0	0
		30	0	0	5.38	8.12	0
		Average	**0.33**	**0**	**1.79**	**2.71**	**0**

*LN, Lymph node. Cut-off T. gondii DNA load: <1 parasite DNA per 1.1 × 10^7^ cells considered as negative (0). D, day post inoculation*.

*Data are presented as the mean number of T. gondii parasite per 1.1 × 10^7^ cells*.

*Average number of parasites is indicated in bold*.

The parasite dissemination from mesenteric LNs to blood was confirmed by assessing the kinetics of the parasite DNA load in PBMCs. In the LR group, the parasite load in PBMCs continued to increase from D4 until its peak at D14. In contrast, in the Gangji infected pigs, parasite DNA was only detected at D8 and D14 post inoculation (Figure 2).

Because *T. gondii* infected immune cells adhesion to lung capillary endothelial cells has been described to trigger parasite egression to immediately coincide in lung tissue and because *T. gondii* cysts preferentially develop in heart tissues during the chronic infection stage (Baba et al., 2017), we also examined the parasite load in heart tissue and highly vascularized lung tissue to confirm the presence of *T. gondii* parasites at this early stage of infection.

**Figure 3:**
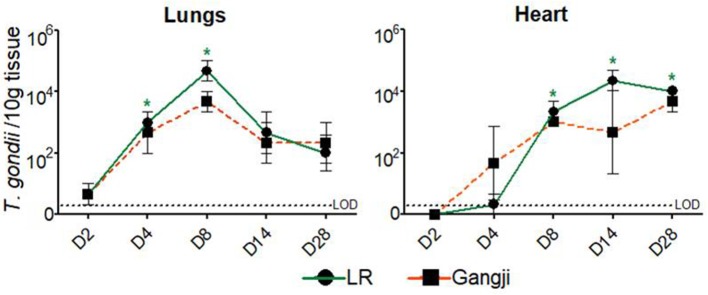
Presence of *T. gondii* DNA in the examined tissues by MCqPCR. The data are presented as the mean ± SD of the number of *T. gondii* parasites/10 g tissue, *significantly different than pre-inoculation, *P < 0.05.

As shown in Figure 3, in lungs, *T. gondii* was already detected in very low amounts at D2, even before being detected in the duodenal lymph nodes, and reached its maximum at D8 in LR (*P* = *0.016*). At day 14 the parasite load decreased to remain less or more stable at D28 in both LR and Gangji groups. In heart, the parasite was detected at D4 in both groups which then steadily increased until reaching its maximum at D14 and D28 in LR (*P* = *0.032*) and Gangji (*P* = *0.095*) groups, respectively (Figure 3). The lymph nodes draining these organs are connected with the thoracic duct in the mediastinum (Riquet et al., 2000). Therefore, the parasite load in immune cells isolated from mediastinal lymph nodes (LNs) might reflect the parasite load in both organs. However, the parasite load in MCs of mediastinal LN slowly increased from D4 to reach its maximum at D8 in both LR (*P* = *0.045*) and Gangji (*P* = *0.0196*) groups and subsequently decreased in a similar manner as observed for the mesenteric LNs of LR infected pigs (Figure 2).

### *T. gondii* Strains Trigger Different Kinetics of Serum IgG Responses

**Figure 4:**
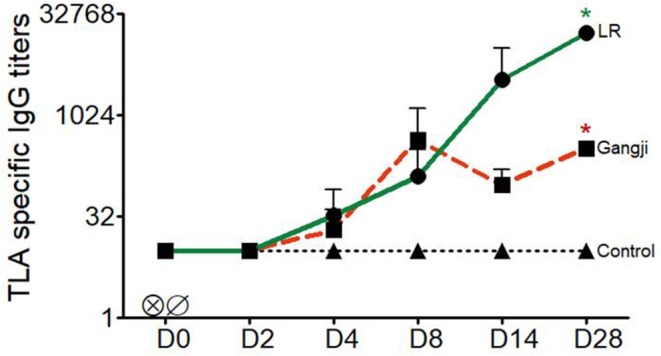
Kinetics of *T. gondii* specific serum IgG titers of LR, Gangji and control groups. Piglets were inoculated orally with tissue cysts of the *T. gondii* IPB LR and Gangji strain on day 0. TLA, *T. gondii* lysate antigens; D, experiment day. The lines are presented as the mean for LR, Gangji and control groups, *LR vs. control, *Gangji vs. control, * or *P < 0.05.

As the LR strain showed earlier and higher loads in lymphoid tissues, we looked if this resulted in a more pronounced TLA-specific IgG response. This was not the case the first week after infection. For both strains low TLA-specific IgG responses could already be detected at D4 after infection, which further increased up until D8, but subsequently started to differ with approximately 50 folds high titer in LR infected pigs at D28, whereas no further increase occurred in the Gangji strain infected pigs (Figure 4) (Supplementary Table 1). Nevertheless, the TLA-specific IgG titers significantly differed at D28 from the pre-infection timepoint in the LR (*P* = *0.015*) and Gangji (*P* = *0.025*) groups.

### *T. gondii* Infection Elicits IFNγ Secreting Immune Cells in Blood and Lymph Nodes

**Figure 5:**
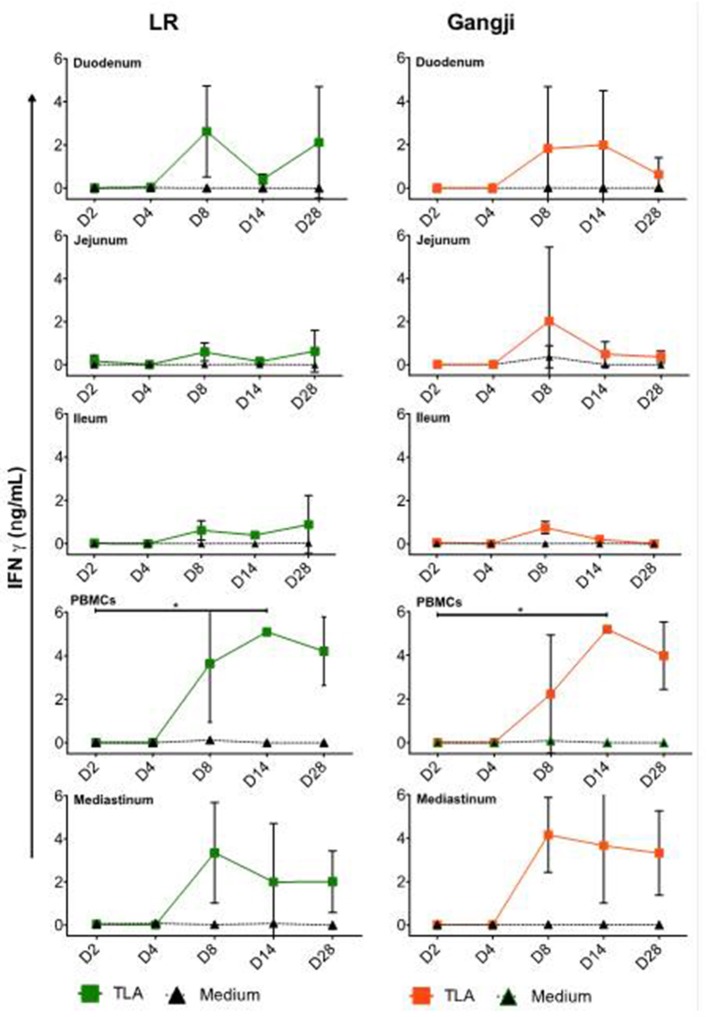
Kinetics of IFNγ secretion by MCs upon oral infection. The data are presented as the mean ± SD, The IFNγ concentration of the control group was below the detection limit and thus was not included. *TLA vs. medium, *P < 0.05.

To be able to correlate the parasite load in the different samples with activation of the cellular immunity, we also assessed the presence of *T. gondii* antigen experienced immune cells in the small intestinal and mediastinal LNs and PBMCs by evaluating their production of IFNγ in an antigen recall assay. As shown in Figure 5, TLA stimulation triggered IFNγ secretion by PBMCs and mediastinal LN cells from D8 onwards for LR and Gangji-infected pigs (Figure 5). IFNγ secretion by mesenteric LN cells was negligible. Unexpectedly, the IFNγ secretion kinetics in both groups was almost equal, which does not correspond with the parasite burden of immune cells and the serum IgG responses.

## Discussion

Oral inoculation of mice with *T. gondii* leads to a primary infection in gut epithelial cells. The *T. gondii* progeny egressing the epithelial cells then infects immune cells in the lamina propria, which subsequently migrate to the adjacent lymph nodes for dissemination in the host (Buzoni-Gatel et al., 2001; Luangsay et al., 2003; Courret et al., 2006; Norose et al., 2008; Baba et al., 2017). Here, we used pigs as an animal model to study infection dynamics and associated immune responses due to the similarity of their gastrointestinal tract with that of humans in terms of physiology, anatomy, immunology and dimensions. We quantified the parasite DNA load in immune cells isolated from duodenal, jejunal, and ileal lymph nodes to be able to assess in which part of the small intestine *T. gondii* first establishes infection. In addition, we included PBMCs to assess the kinetics of systemic spread and cells from mediastinal lymph nodes, as the latter drain the heart, one of the most parasitized tissues after brain during *T. gondii* infection (Gisbert Algaba et al., 2017).

The LR strain showed a more pronounced replication in pigs than the Gangji strain, as evidenced by an earlier detection in duodenal mesenteric LNs, a longer persistence in the mesenteric LNs and blood and a higher antibody response upon infection. The reason might be that the LR strain was isolated from pigs and therefore is more adapted to infect pigs, whereas the human isolated Gangji strain is not and might need to adapt to efficiently infect pigs. A similar phenomenon was for instance observed for influenza as human species/strains need to adapt to pigs to establish efficient infection (Rajao et al., 2019). That *T. gondii* DNA in mononucleated cells (MCs) of duodenal LNs was first detected at day 4 post inoculation, indicates that upon excystation the *T. gondii* LR strain might first infect the duodenum. On the other hand, in the Gangji group, the parasite load in PBMCs and MLNs was below the detection limit until D8. Moreover, as *T. gondii* DNA was detected in blood and lymph node immune cells, this seems to indicate that the dissemination from the intestine to the lymph nodes and other organs in pigs is at least in part immune cell mediated and might occur via a “Trojan horse” mechanism, as described for *T. gondii* dissemination in mice (Courret et al., 2006; Sanecka and Frickel, 2012). However, this finding requires further investigation as the presence of viable parasites in immune cells was not assessed.

In addition to lymph node and blood immune cells, we also assessed the parasite load in lungs and heart. Surprisingly, the parasite load in lung tissue of the LR and Gangji strain-infected groups was almost equal despite the more pronounced replication of LR in mesenteric LN and blood. Interestingly, *T. gondii* was detected at day 2 post inoculation in lungs, even before we could detect it in duodenal LNs, indicating that the parasite quickly disseminates from its initial point of entry in the gut to the lungs. A potential route might be via the pancreatic LNs, which drain both pancreas and cranial duodenum or directly via the inferior pancreaticoduodenal veins. Alternatively, since we orally inoculated the pigs, we cannot exclude a mis-direction of some inoculant into the respiratory tract.

In mice, *T. gondii* is able to infect vascular endothelial cells and use these cells as a replication niche for dissemination to other organs (Gregg et al., 2013; Konradt et al., 2016). Our results seem to indicate that porcine lung cells support *T. gondii* replication. This could be explained the oxygen levels in the tissue. In most tissues, *T. gondii* requires activation of hypoxia-inducible factor 1 (HIF1) for replication at physiologically relevant oxygen levels (3%). In lung tissue, HIF1 activation is not required as oxygen levels range between 4 and 14% (Spear et al., 2006). However, further studies are needed to confirm *T. gondii* stage conversion from rapidly multiplying tachyzoites to the slow replicating bradyzoites and to investigate if the replication in the lungs might result in the formation of tissue cysts during later stages of infection, as *T. gondii* DNA did not increase beyond day 8 post inoculation. The parasite load in heart tissue on the other hand continuously increased from day 4 onward and remained high until the end of the experiment in both groups. These results agree with the fact that the heart is indeed the most parasitized organ in early *T. gondii* infection stage irrespective of the strain (Jennes et al., 2017). Based on the profile in heart tissue, we assume that we should have detected consistent *T. gondii* DNA in the mediastinal LN after D8; however, we did not. This seems to indicate that there is drainage to other lymph nodes, such as- peritracheobronchial lymph nodes (Riquet et al., 2000).

In addition to the quantification of the parasite load in immune cells and tissues, antibody and cellular immune responses were evaluated in LR and Gangji infected groups over time as well. A robust serum IgG response was detected in both LR and Gangji groups. However, the serum IgG response in the LR group was stronger than in the Gangji group, which corresponds to our previous study (Jennes et al., 2017). We also assessed IFNγ secretion by blood and lymph node immune cells of both groups. As expected from our previous research, IFNγ secretion was detected in PBMCs at D14 in both LR and Gangji groups, indicating the presence of peripheral antigen experienced T cells in both groups (Jennes et al., 2017; Rahman et al., 2019). For the lymph nodes, immune cells from mediastinal and duodenal lymph nodes secreted IFNγ, while jejunal and ileal lymph node immune cells did not secrete IFNγ upon TLA re-stimulation. This result further supports that *T. gondii* first infects duodenal epithelial cells. Unexpectedly, a similar IFNγ secretion profile was observed for immune cells isolated from Gangji infected pigs, although the LR strain consistently showed a higher parasite DNA load in these tissues and a higher antibody response than the Gangji strain. A better host adaptation of the LR strain to pigs might explain this. In mice, tachyzoite proliferation of a *T. gondii* strain has been related to IFNγ-inducible cytoplasmic effector proteins, the 47 kDa immunity-related GTPases (IRG proteins). These proteins can inhibit proliferation. Some *T. gondii* strains secrete kinases and pseudokinases that can inactivate IRG proteins resulting in increased replication. Adaptation of the *T. gondii* strain might have resulted in sufficient overriding of the IRG control mechanism to allow more replication (Lilue et al., 2013). A more thorough genetic comparison of both strains might confirm this. Whether this is due to genotype differences between both strains, warrants further investigation. It would be interesting to study the efficiency of both strains at invading intestinal epithelial cells and undergoing replication via an *in vitro* plaque assay (Di Cristina et al., 2017). This would provide some information as to whether the higher parasite load of the LR strain is due to the host response or immune escape mechanisms of the LR strain.

Taking these results into account we assume that the parasite burden in the small intestine is related to the serum antibody responses. In the LR group, high parasite loads in the gut correspond to high serum IgG responses, while in the Gangji group low intestinal parasite loads correspond to low serum IgG responses. Although this seems to contradict the parasite load in the tissues, we speculate that the significant antibody responses in the LR group restrict dissemination to the target organs, while in the Gangji group dissemination is less restricted. This might explain the almost equal parasite load in heart and lungs and the similar T cell responses in both groups.

In conclusion, pigs serve as an interesting model to study initial *T. gondii* infection kinetics in the gut, the associated immune responses and the subsequent dissemination to organs. Our data indicate that upon ingestion *T. gondii* first enters the host at the duodenum and then disseminates to other tissues. This is associated with the activation of IFNγ secreting immune cells. However, it does not yet explain why a re-infection with the Gangji strain in LR strain infected pigs cleared the *T. gondii* DNA from tissue. Nevertheless, these findings lay a foundation to further study the early stages of *T. gondii* intestinal infection and might inform on strategies to prevent initial invasion of the host by this parasite.

## Data Availability Statement

All datasets generated for this study are included in the article/Supplementary Material.

## Ethics Statement

The animal procedures were approved by the Ethical Committee (EC) of the Faculty of Veterinary Medicine and the Faculty of Bioscience Engineering, Ghent University (EC 2009/149) and by the EC of Sciensano, Belgium (176 20140704-01).

## Author Contributions

MR, MJ, and EC designed the study. MR and MJ performed the experiments. MR acquired and analyzed the data and drafted the manuscript. BD analyzed the data and wrote the manuscript. IG performed MCqPCR and revised the manuscript. PD and KD helped to design the study, gave valuable input, and revised the manuscript. SD revised the manuscript. EC analyzed the data and reviewed the manuscript.

### Conflict of Interest

The authors declare that the research was conducted in the absence of any commercial or financial relationships that could be construed as a potential conflict of interest.

## Abbreviations

ELISA: Enzyme-Linked ImmunoSorbent Assay
IFA: Indirect Immunofluorescence Assay
MAT: Modified Agglutination Test
MC-qPCR: Magnetic Capture Quantitative Polymerase Chain Reaction
Cp: Crossing point (also known as Ct or Cq)
PBS: Phosphate-buffered saline
ng/ml: Nanogram/milliliter
TLA: Total Lysate Antigen
FACS: Fluorescence-activated cell sorting
IFN-γ: Interferon gamma
PBMCs: Peripheral blood mononuclear cells
LOD: Limit of detection
SD: Standard deviation
CD: Cluster of differentiation
NK cells: Natural killer cells
IL: Interleukin
APCs: Antigen presenting cells
CTLs: Cytotoxic T lymphocytes
Th cells: T helper cells.
