# Supplementary material for: Early Kinetics of Intestinal Infection and Immune Responses to Two Toxoplasma gondii Strains in Pigs
Source: Front Cell Infect Microbiol. 2020 Apr 16;10:161. doi: 10.3389/fcimb.2020.00161 (PMC7176905; doi:10.3389/fcimb.2020.00161)
Supplement: Supplementary file 1 [file Table_1.docx]

**Supplementary Table 1:** *T. gondii*-specific serum IgG titers of LR, Gangji and control groups.

| **Timepoint** | **IgG titers** | | | | | | | | |
| --- | --- | --- | --- | --- | --- | --- | --- | --- | --- |
|  | **LR** | | | **Gangji** | | | **Control** | | |
|  | Pig 1 | Pig 2 | Pig 3 | Pig 1 | Pig 2 | Pig 3 | Pig 1 | Pig 2 | Pig 3 |
| D0 | <10 | <10 | <10 | <10 | <10 | <10 | <10 | <10 | <10 |
| D2 | <10 | <10 | <10 | <10 | <10 | <10 | <10 | <10 | <10 |
| D4 | <10 | <10 | 80 | 40 | <10 | <10 | <10 | <10 | <10 |
| D8 | 20 | 320 | 40 | 20 | 1280 | 20 | <10 | <10 | <10 |
| D14 | 20 | 20 | 10240 | 80 | 160 | 40 | <10 | <10 | <10 |
| D28 | 20480 | 20480 | 10240 | 320 | 320 | 340 | <10 | <10 | <10 |

Limit of detection: titer <10
